# Supplementary material for: Agonist-induced dimer dissociation as a macromolecular step in G protein-coupled receptor signaling
Source: Nat Commun. 2017 Aug 9;8:226. doi: 10.1038/s41467-017-00253-9 (PMC5548745; doi:10.1038/s41467-017-00253-9)
Supplement: Supplementary file 1 — Supplementary information [file 41467_2017_253_MOESM1_ESM.pdf]

Title of file for HTML: Supplementary Information
Description: Supplementary notes, supplementary figures

Title of file for HTML: Peer Review File
Description:

Supplementary Note 1

List of abbreviations:

dcFRAP	-	double color fluorescence recovery after photobleaching
CRISPR	-	clustered regularly interspaced short palindromic repeats
CD	-	circular dichroism
DDM	-	*n*-Dodecyl β-D-maltoside
DVL	-	Dishevelled
ERK1/2	-	extracellular signal-regulated kinase1/2
FCCS	-	fluorescence cross correlation spectroscopy
FZD	-	Frizzled
ITC	-	isothermal titration calorimetry
GPCR	-	G protein-coupled receptor
MLE-12	-	mouse lung epithelial cell line
SEC	-	size exclusion chromatography
TM1, 4, 5	-	transmembrane spanning helices 1, 4, 5
TSS	-	transcription start site
WNT	-	WNT/Int-1 family of proteins

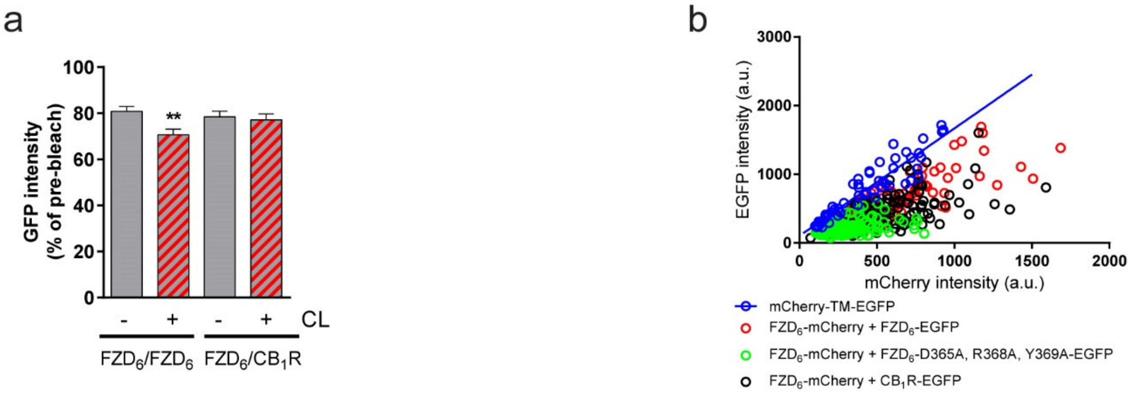

c

FZD6-GFP	FZD6-mCherry	K	CB1 assumed	RMS error	Comment
dimer (xg=2)	dimer (xr=2)	0,17	monomer	0,12	best fit, realistic K value
dimer (xg=2)	dimer (xr=2)	0,34	dimer	0,29	
monomer (xg=1)	monomer (xr=1)	0,34	monomer	0,31	
monomer (xg=1)	monomer (xr=1)	0,68	dimer	0,09	poor fit, unrealistically high K

Supplementary Figure 1: Controls and measures of FZD_6 expression levels and dimer stoichiometry. (**a**) Statistical analysis of data from **Fig. 1d, g** by one-way ANOVA to support the specificity of the FZD_6/FZD_6 dimerization over unspecific interactions in the presence of CB_1. The mobile fractions for FZD_6-GFP and CB_1-GFP when co-expressed with V5-FZD_6-mCherry are compared in the presence and absence of anti-V5/avidin crosslinking (CL). Error bars provide s.e.m. ** P<0.01. (**b**) V5-FZD_6-mCherry was present in excess relative to FZD_6-GFP (red), (black) or FZD_6-GFP dimer mutant (green); related to Fig. 1, 2. Fluorescence intensity values of the mCherry-TM-GFP construct (also called “stoichiometer”) were quantified with identical acquisition settings as used in the dcFRAP experiments (blue). Values deemed to be appropriate for the analysis fell below the line. Data for the control construct were fit to a linear least-squares model (slope=1.6; R^2= 0.8739; N=54) and this line is shown superimposed. (**c**) Corresponding to the data presented in **Fig. 1j** assessing the FCCS data from FZD_6-GFP/V5-FZD_6-mCherry and FZD_6-GFP/CB_1-mCherry experiments, results for the different model fits are presented in table form. The model fit is assessed for data assuming that FZD_6 forms dimers (row 1, 2) or monomers (row 2, 3) in combination with the assumption that CB_1 forms monomers or dimers. K is the crosstalk parameter. RMS described the agreement with the theoretical model. See also Method section for detailed information.

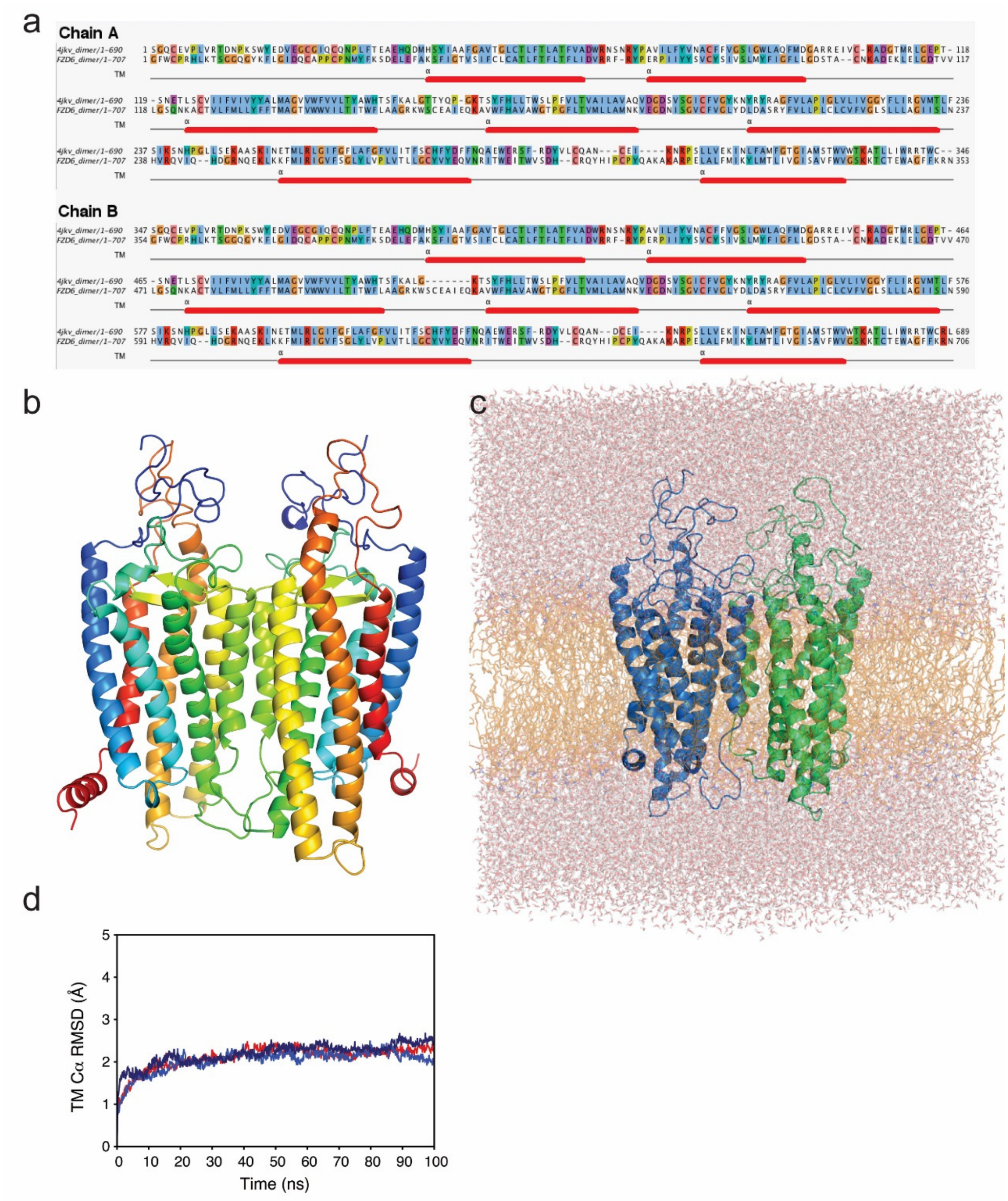

Supplementary Figure 2: Homology model of FZD6, related to Figure 2. (**a**) Sequence alignment used for homology modeling of the FZD6 homodimer with the crystallographic structure of SMO (PDB code 4JKV) as a template. Membrane-spanning helices according to the SMO structure are annotated by red cylinders. (**b**) FZD6 homodimer model obtained based on the high resolution crystal structure of SMO. Rainbow coloring, N terminus – blue; C terminus – red. (**c**) Representation of the membrane-inserted and equilibrated FZD6 dimer model. Secondary structures are represented as cartoons and individual protomers are colored in blue and green. POPC lipids and water molecules are represented as sticks with orange carbon and red oxygen sticks, respectively. (**d**) Structural integrity, measured as RMSD of Cα atoms of the TM regions to the initial model, for three independent molecular dynamics simulations of the FZD6 dimer model.

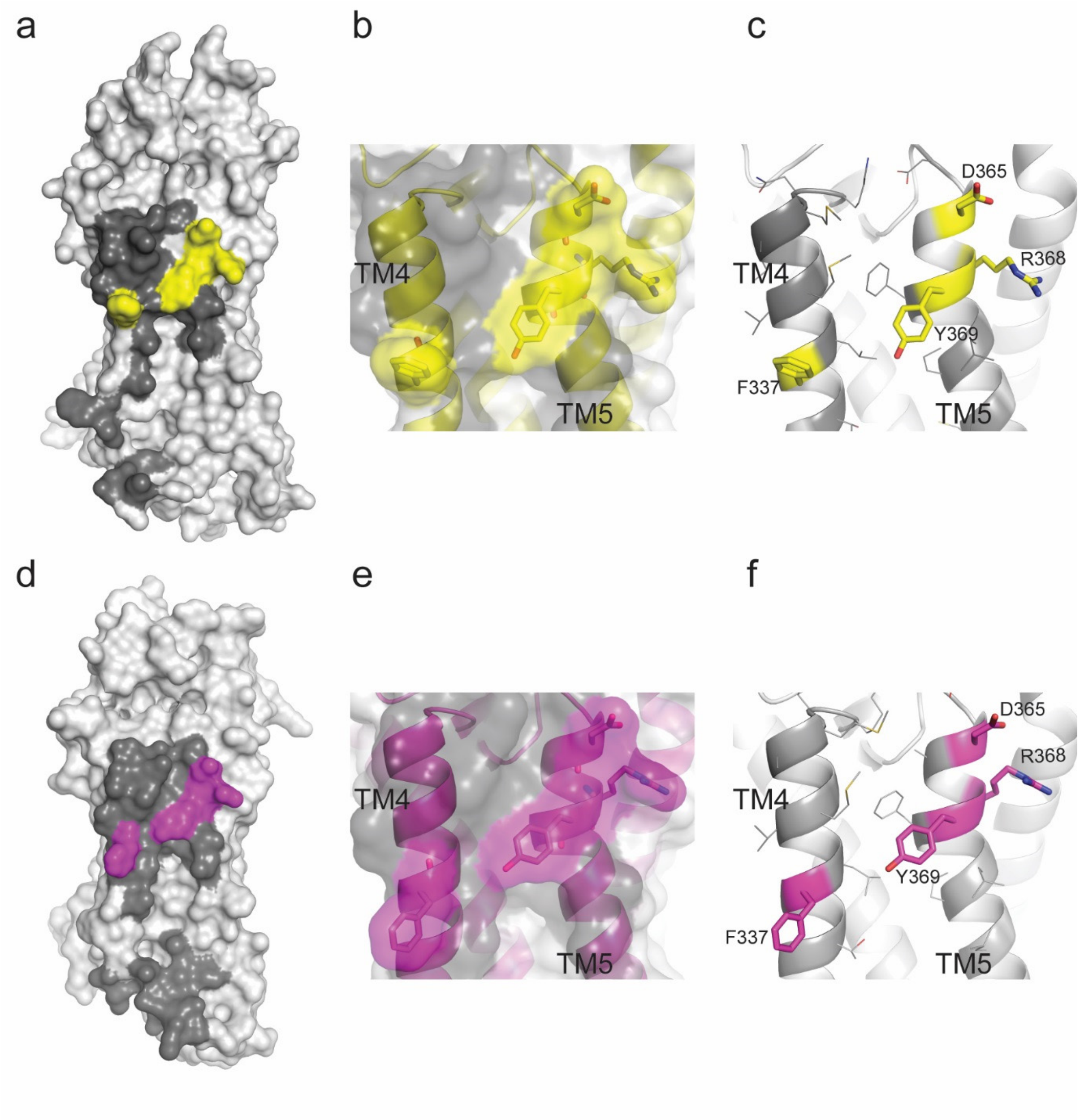

Supplementary Figure 3: Space-filling models, backbone and ribbon diagrams of the FZD_6 protomers, related to Figure 2. The magnified images provide a more detailed view of the proposed dimer interface of FZD_6. According to Fig. 2D in the main text, the two protomers of the dimer are shown in **a** and **d** as space filling models. Panel **b**, **c** and **e**, **f** show the residues in the dimer interface evaluated by mutagenesis. The dark grey surface represents the proposed interface while color-coded surfaces (yellow/magenta) represent the mutations in TM4 (F337) and TM5 (D365, R368, Y369) that were experimentally shown to negatively affect FZD_6 dimerization.

Mutant		Dimer	Subcellular localization	GFP-tagged mutant in HEK293 cells	dcFRAP (85-101s)
FZD6-wt	wild type	Yes	Membraneous	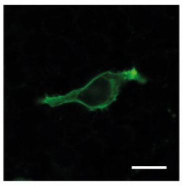	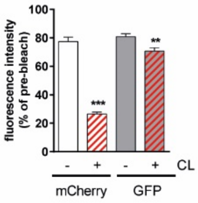
FZD6-D365A, R368A, Y369A	dimer mutant	No	Membraneous	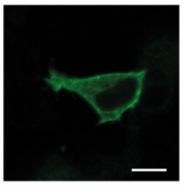	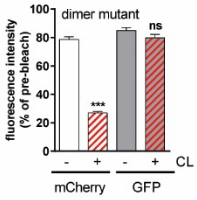
FZD6-D365E, R368K, Y369F	dimer control mutant	Yes	Membraneous	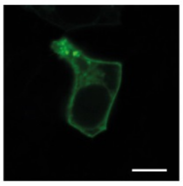	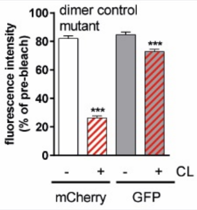
FZD6-Y369A		No	Membraneous	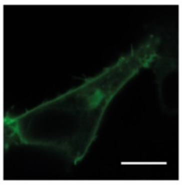	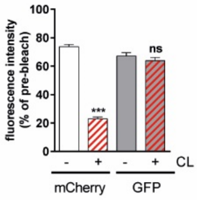
FZD6-F337A		No	Membraneous	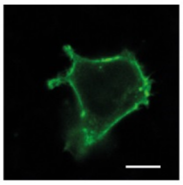	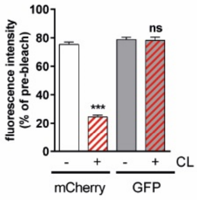
FZD6-M341A, M345A		Yes	Membraneous	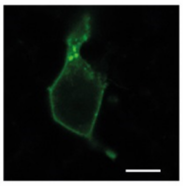	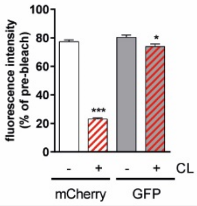
FZD6-M341A, M345A, N346A		N.D.	Intracellular	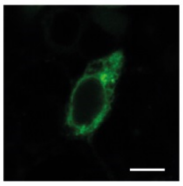	not applicable

Supplementary Figure 4: Mutational analysis of FZD6, related to Figure 2. The table summarizes information about FZD6 mutants employed in this study with regard to mutated amino acids, the ability to dimerize and their respective expression patterns.

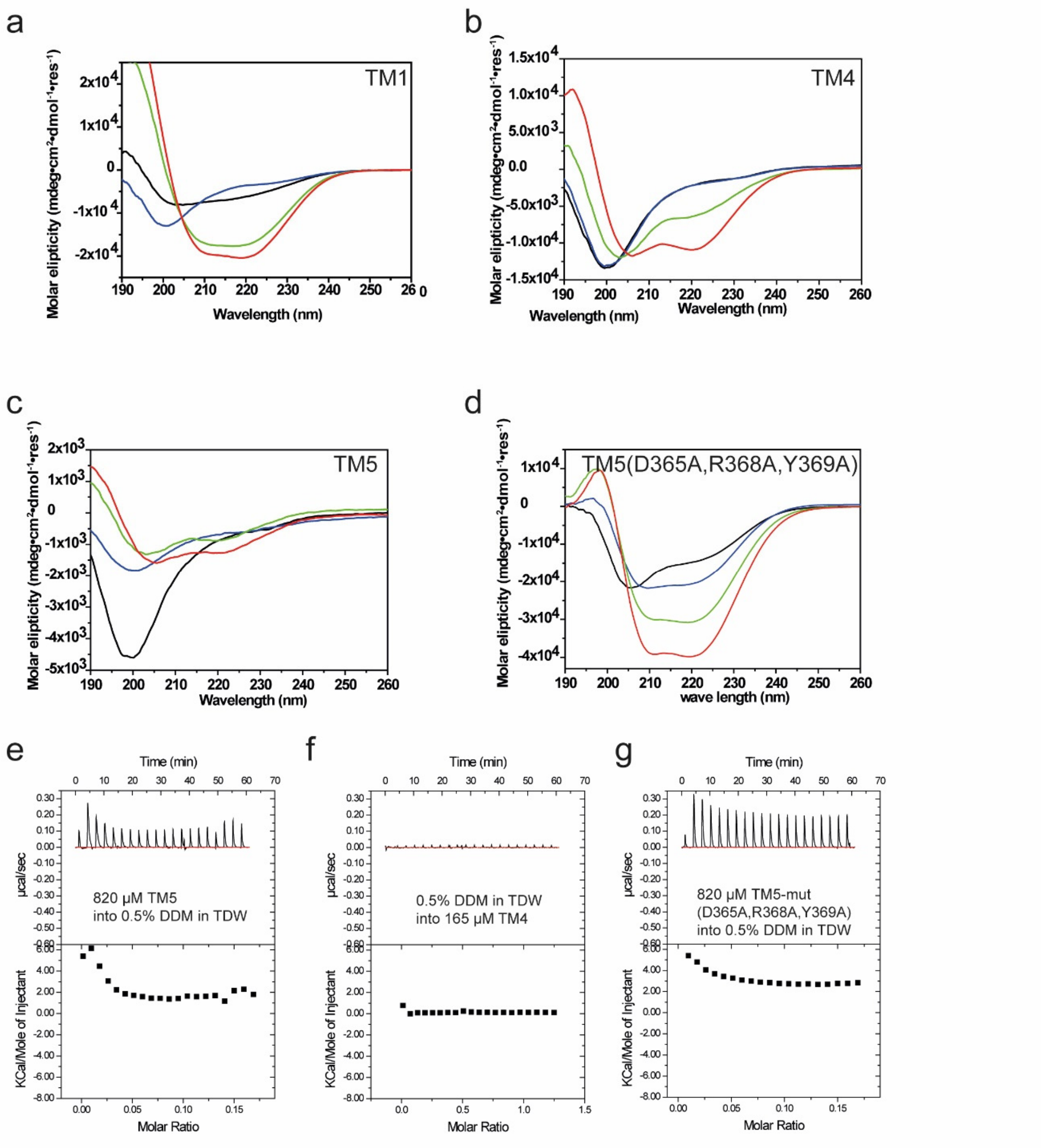

Supplementary Figure 5: Circular dichroism (CD) and isothermal titration calorimetry (ITC) experiments for structural analysis of synthetic peptides resembling TM1, 4, 5 and mutant TM5 and of peptide-peptide interactions, related to Figure 3. Synthetic peptides (for sequence see **Supplementary Fig. 9**) resembling TM1 (**a**), 4 (**b**), 5 (**c**) and mutant TM5 (**d**) were used for CD experiments in triple distilled water with increasing DDM concentrations (0% DDM – black, 0.01% DDM – blue, 0.1% DDM – green and 0.5% DDM – red). (**c**)TM5 is unstructured in TDW and begins to gain structure at 0.1% DDM. It forms a clear α-helical structure in 0.5% DDM. The triple mutated TM5 (**d**) forms an α-helix in a much lower DDM concentration (0.01% DDM). This indicates that the mutations induce a more stable α-helical structure of the peptide. (**e-g**) Isothermal titration calorimetry (ITC) experiments in **e-g** serve as control for data provided in **Fig. 3**: Titration of (**e**) 820 µM TM5 into 0.5% DDM in TDW, (**f**) 0.5% DDM in TDW into 165 µM TM4 and (**g**) 820 µM TM5 mut into 0.5% DDM in TDW.

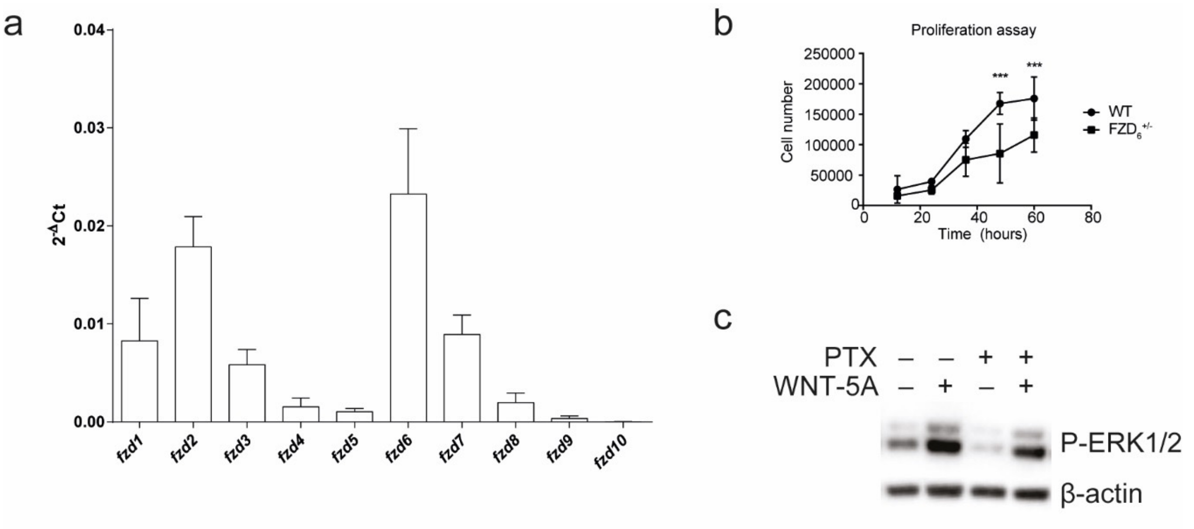

Supplementary Figure 6: FZD$_6$ in mouse lung epithelial cells, related to Figure 6. (**a**) Expression profile of *fzd1-10* mRNA in mouse lung epithelial cells (MLE-12). The bar graph depicts expression levels of *fzd1-10* mRNA in mouse lung epithelial cells (MLE-12) measured by quantitative PCR. Error bars provide standard s.e.m. N=3. Primer efficiency has been quantified previously (Halleskog et al., 2012). (**b**) MLE-12 wt and CRISPR/Cas9 FZD$_6^{+/-}$ cells were seeded in a 24-well plate and counted after trypsinization at the indicated time points. Cell proliferation was quantified by cell counting in a Bürker chamber. Column factor: P=0.0003: F (1, 20) =19.49. Row factor: P < 0.0001: F (4, 20)=29.69. *** P<0.001 (two-way ANOVA). Error bars give s.e.m. (**c**) MLE-12 cells were seeded in a 24-well plate. The next day cells were starved, treated with PTX (100 ng ml^{-1}) overnight and then stimulated for 2.5 min with 300 ng ml^{-1} WNT-5A. Cell lysates were analyzed for P-ERK1/2 and β-actin levels by immunoblotting. The WNT-5A-mediated ERK1/2 phosphorylation is partially sensitive to PTX suggesting that the response is dependent on G$\alpha_{i/o}$ and PTX-insensitive Gα_q proteins, in agreement with previous findings that FZD$_6$ is a G$\alpha_{i1/q}$-coupled receptor (Kilander et al., 2014).

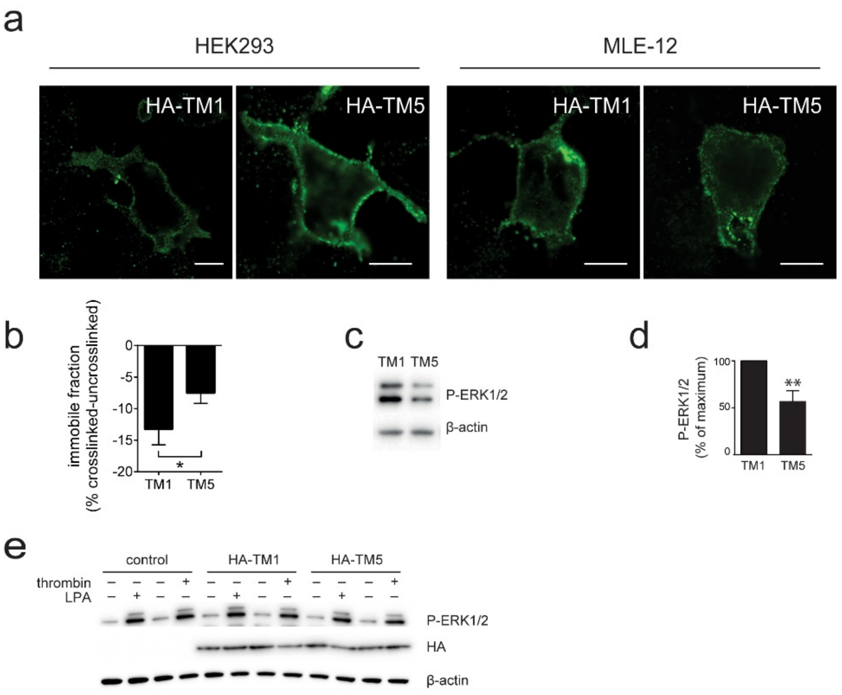

Supplementary Figure 7: Control experiments for assaying receptor dimerization by dcFRAP and expression of minigenes. (**a**) HEK293 and MLE-12 cells were transfected with HA-tagged TM1/5 minigenes, fixed in 4% paraformaldehyde and stained with an anti-HA antibody/Cy2-conjugated secondary antibody in the absence of cell permeabilization by detergents. Positive anti-HA staining in the cell membrane confirms both the membrane expression of the minigenes as well as the correct orientation of the peptides with the N-terminal signal sequence/HA tag on the extracellular side accessible to the primary antibody. Images were taken with a 40x objective using the 488 nm laser line on a Zeiss LSM 710 confocal microscope. Size bars – 10 µm. (**b**) Bar graph compares the immobile fraction in dcFRAP experiments of FZD_6-GFP after CL of V5-FZD_6-mCherry in cells expressing minigenes corresponding to either TM1 (control) or TM5 (competing at the dimer interface). P= 0.0472; t=2.008; df=107; N=43 TM1; N=66 TM5 from 4-5 independent experiments). Error bars give s.e.m.; * P<0.05 (two-tailed t-test). (**c**) Transient transfection of TM5, but not TM1 minigenes in MLE-12 cells reduced basal P-ERK1/2 indicating that the abrogation of dimer formation, in the presence of TM5, reduces receptor-mediated signaling. (**d**) Bar graph summarizes four independent immunoblotting experiments. P-ERK1/2 in TM1 expressing MLE-12 cells were normalized to 100%. P= 0.0091; t=3.791; df=6; N=4). Error bars give s.e.m.; ** P<0.001 (two-tailed t-test). Quantification of P-ERK1/2 is normalized to β-actin as loading control. (**e**) HEK293 cells were transfected with empty vector or minigenes encoding either HA-TM1 or HA-TM5, serum-deprived and then stimulated with either lysophosphatidic acid (10 µM) or thrombin (10 U/ml) for 10 min. P-ERK1/2, HA-TM1/5 and β-actin as loading control were assessed by PAGE/immunoblotting. Presence of TM1 or TM5 does not affect agonist-induced ERK1/2 phosphorylation mediated by endogenously expressed Class A GPCRs (lysophosphatidic acid receptor, protease-activated receptor).

Uncropped immunoblots for Figure 6a

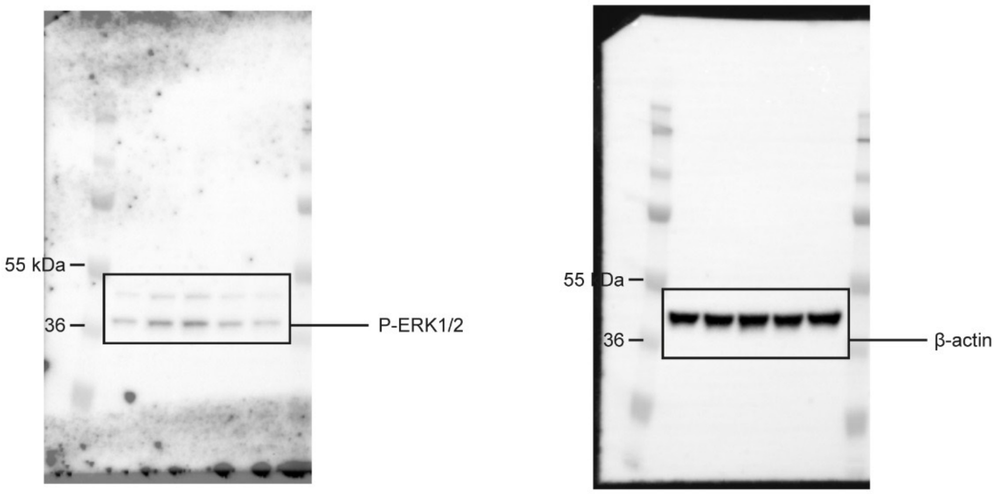

Uncropped immunoblots for Figure 6c

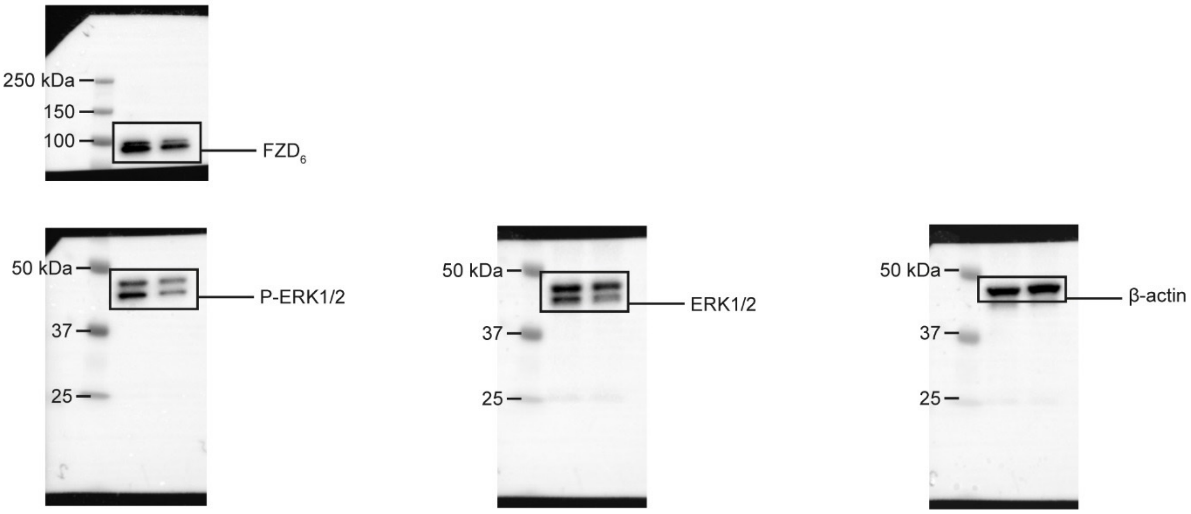

Supplementary Figure 8

Uncropped immunoblots for Figure 6d

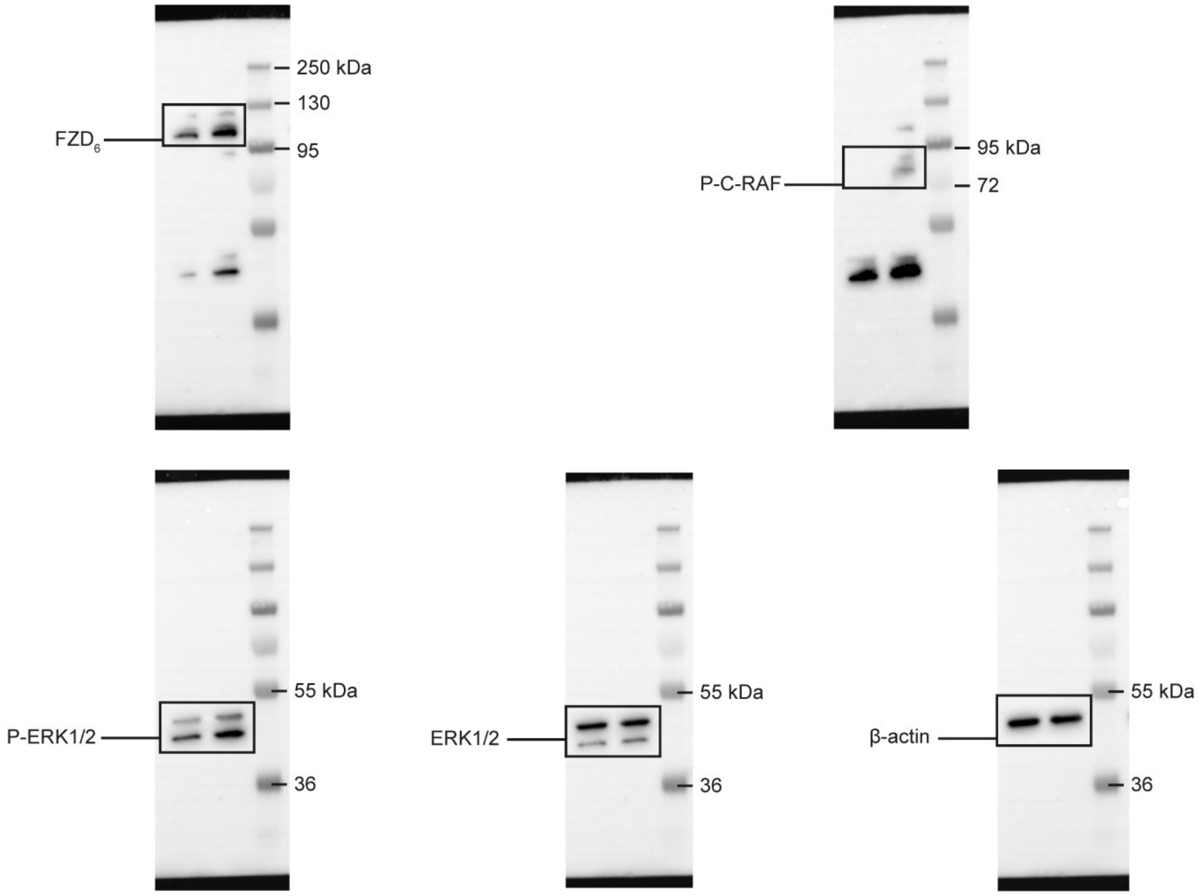

Uncropped immunoblots for Figure 6e

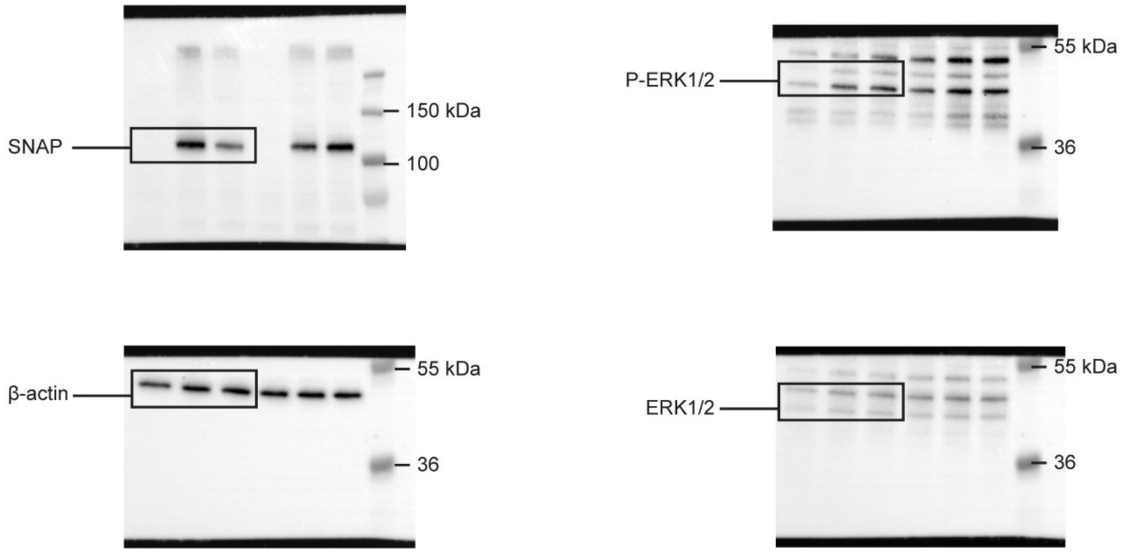

Supplementary Figure 9

Uncropped immunoblots for Figure 6f

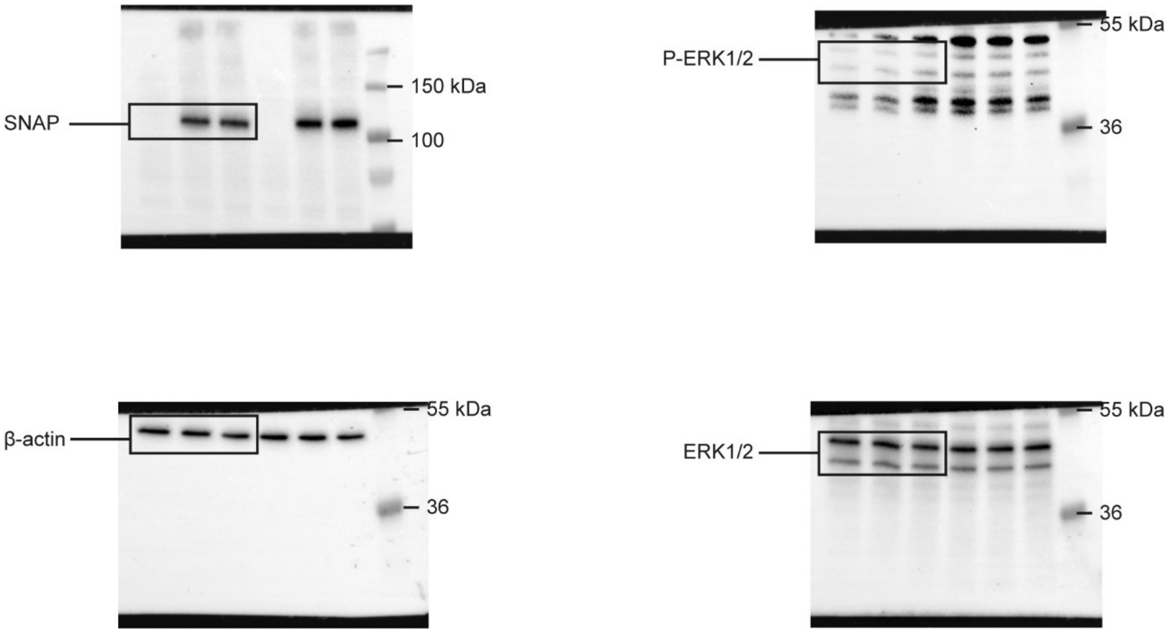

Supplementary Figure 10
